# Evaluation of Conventional and Combined Doppler Parameters in Preeclampsia: Diagnostic and Prognostic Insights

**DOI:** 10.3390/jcm14020647

**Published:** 2025-01-20

**Authors:** Gulsan Karabay, Burak Bayraktar, Zeynep Seyhanli, Ahmet Arif Filiz, Betul Tokgoz Cakir, Gizem Aktemur, Nazan Vanli Tonyali, Recep Taha Agaoglu, Gulcan Kocaoglu, Umut Karabay, Kadriye Yakut Yucel

**Affiliations:** 1 Department of Obstetrics and Gynecology, Division of Perinatology, Ankara Etlik City Hospital, Ankara 06170, Turkey; drburakbayraktar@gmail.com (B.B.); drzeynepseyhanli@gmail.com (Z.S.); ahmetarif35.filiz@gmail.com (A.A.F.); btltkgz@gmail.com (B.T.C.); drgizemkizilbuga@gmail.com (G.A.); nazanvanli@gmail.com (N.V.T.); tahaagaoglu@hotmail.com (R.T.A.); yakutkadriye@hotmail.com (K.Y.Y.); 2 Department of Obstetrics and Gynecology, Ankara Etlik City Hospital, Ankara 06170, Turkey; gulcankocaoglu@gmail.com; 3 Department of Internal Medicine, Gulhane Training and Research Hospital, Ankara 06010, Turkey; umut_karabay@hotmail.com

**Keywords:** preeclampsia, doppler, ultrasonography, cerebroplacental ratio, cerebral–placental–uterine ratio, umbilical-to-cerebral ratio, amniotic-to-umbilical-cerebral ratio

## Abstract

**Background**: The aim of this study was to examine the relationship between conventional and novel Doppler parameters, including cerebroplacental ratio (CPR), cerebral–placental–uterine ratio (CPUR), umbilical-to-cerebral ratio (UCR), and amniotic-to-umbilical-cerebral ratio (AUCR), with the diagnosis of preeclampsia (PE) and adverse neonatal outcomes in PE cases. **Methods**: This prospective case-control study was conducted at the Ankara Etlik City Hospital Perinatology Clinic between November 2023 and May 2024. The study population was divided into two groups: Group 1, consisting of 74 patients diagnosed with preeclampsia, and Group 2, consisting of 80 healthy control patients. Composite adverse perinatal outcomes (CANOs) include presence of at least one adverse outcome: 5th-minute APGAR score < 7, transient tachypnea of the newborn (TTN), respiratory distress syndrome (RDS), need for continuous positive airway pressure (CPAP), need for mechanical ventilation, neonatal intensive care unit (NICU) admission, neonatal hypoglycemia, need for phototherapy, intraventricular hemorrhage (IVH), and neonatal sepsis. **Results**: The CPR, CPUR, and AUCR were significantly lower in the PE group compared to the control group, while the UCR was notably higher in the PE group. Among the combined ratios, the CPUR exhibited the highest diagnostic performance for both PE diagnosis and the prediction of CANOs. Additionally, while the UCR, CPR, and AUCR were significant for PE diagnosis, only AUCR demonstrated a significant association with the prediction of CANOs. **Conclusions**: Combined Doppler parameters, especially CPUR and AUCR, offer valuable insights into diagnosing PE and predicting CANOs. CPUR demonstrated the highest diagnostic accuracy, underscoring its potential utility in clinical settings.

## 1. Introduction

Preeclampsia (PE) is a significant obstetric complication occurring after the 20th week of pregnancy, affecting 2–8% of pregnant women globally [1,2]. It remains one of the leading causes of maternal and fetal morbidity and mortality. Maternal complications include placental abruption, pulmonary edema, and acute renal failure, while fetal complications range from fetal growth restriction (FGR), preterm birth, low Apgar scores, the need for neonatal intensive care, and even fetal death [1].

Although the pathogenesis of PE is not yet fully understood, a defect in placental invasion is recognized as a central issue [3,4]. Abnormal placental invasion, combined with endothelial dysfunction and maternal vasoconstriction, leads to placental hypoxia. Understanding fetal adaptations to this developing placental hypoxia is critical for evaluating fetal well-being and optimizing clinical management. Doppler ultrasonography is the most commonly used non-invasive method in clinical practice for predicting uteroplacental insufficiency, fetal well-being, and the course of pregnancy [5,6]. Conventional Doppler parameters including measurements of the uterine artery (UtA), middle cerebral artery (MCA), and umbilical artery (UA) systolic/diastolic (S/D) ratios, and pulsatility indices (PIs). However, the predictive value of these parameters for placental and fetal hypoxia and composite adverse neonatal outcomes (CANOs) remains limited. In recent years, combined Doppler parameters have gained attention for their potential to enhance diagnostic accuracy [7,8,9,10]. These combined parameters include the cerebroplacental ratio (CPR), cerebral–placental–uterine ratio (CPUR), umbilical-to-cerebral ratio (UCR), and amniotic-to-umbilical-cerebral ratio (AUCR), and to our knowledge, UCR and AUCR have not been previously investigated in PE. The CPR, calculated as the ratio of MCA PI to UA PI, has been strongly associated with adverse neonatal outcomes, particularly in FGR cases [7]. The CPUR, calculated as the ratio of CPR to UtA PI, has demonstrated associations with fetal well-being [8]. The UCR (UA PI to MCA PI) and AUCR (single deepest pocket to UCR) are relatively new combined ratios and have been identified as important parameters to evaluate fetal well-being [9,10].

Identifying pregnant women at high risk of maternal and fetal complications in PE is critical for effective patient monitoring, postnatal maternal–fetal care, birth planning, and minimizing unnecessary obstetric interventions [11]. The aim of this study was to examine the relationship between conventional and novel Doppler parameters, including UCR and AUCR, and the diagnosis of PE and adverse neonatal outcomes in PE cases. The study aimed to compare the predictive power of these Doppler parameters in diagnosing PE and predicting adverse neonatal outcomes.

## 2. Materials and Methods

This prospective case-control study was conducted at the Ankara Etlik City Hospital Perinatology Clinic between November 2023 and May 2024. The study population was divided into two groups: Group 1, consisting of 74 patients diagnosed with preeclampsia (39 with early-onset preeclampsia (EOPE) and 35 with late-onset preeclampsia (LOPE)), and Group 2, consisting of 80 healthy control patients. The study protocol was approved by the Ethics Committee of Ankara Etlik City Hospital (approval number: AESH-EK1-2023-622). All participants were informed about the study, and written consent was obtained. The study was conducted in accordance with the principles outlined in the Declaration of Helsinki.

PE was diagnosed based on the criteria established by the American College of Obstetricians and Gynecologists (ACOG) [12]. Diagnosis required a systolic blood pressure of ≥140 mm Hg or diastolic blood pressure of ≥90 mm Hg, measured on two occasions at least 4 h apart after the 20th week of pregnancy, accompanied by proteinuria. Proteinuria was defined as ≥300 mg in a 24-h urine sample, a protein/creatinine ratio of ≥0.3 in a spot urine sample, or 2+ protein on dipstick testing. In the absence of proteinuria, PE was also diagnosed if hypertension was accompanied by any of the following criteria: platelet count <100,000/µL; serum creatinine >1.1 mg/dL or a doubling of serum creatinine levels without other renal disease; elevated liver transaminases to twice the normal level; pulmonary edema; or a persistent headache with unexplained neurological symptoms. PE is further classified into two subtypes based on the timing of onset. EOPE occurs before 34 weeks of gestation, while LOPE occurs at or after 34 weeks of gestation [13,14]. This classification reflects the distinct pathophysiological mechanisms and clinical implications of EOPE and LOPE, offering valuable guidance for diagnosis and management strategies.

The gestational age of all participants was confirmed through ultrasound measurements of crown–rump length taken between 11 and 14 weeks of gestation. Exclusion criteria included patients with chronic maternal diseases (e.g., diabetes, thyroid dysfunction), smoking or alcohol consumption, congenital fetal anomalies, and obstetric complications other than PE, such as FGR or gestational diabetes mellitus.

Demographic data were collected from all participants, including maternal age, weight, weight gain during pregnancy, body mass index (BMI), and previous pregnancy history (gravida, parity). Maternal venous blood samples were analyzed to measure hemoglobin level, platelet count, aspartate aminotransferase (AST), alanine aminotransferase (ALT), albumin, uric acid, and fibrinogen levels. Ultrasound examinations were performed and documented by a maternal–fetal medicine expert using a Voluson S10 Expert sonography machine (GE Healthcare, Milwaukee, WI, USA) via transabdominal ultrasound. Fetal ultrasonographic measurements included the single deepest pocket (SDP) for amniotic fluid and Doppler parameters such as UtA S/D ratio and PI, MCA S/D ratio and PI, and UA S/D ratio and PI. All measurements were conducted in accordance with the protocols established by the International Society of Ultrasound in Obstetrics and Gynecology (ISUOG) [15]. The CPR was calculated as the ratio of MCA PI to UA PI [7]. The CPUR was derived by dividing the CPR by UtA PI [8]. The UCR was determined as the inverse of CPR, calculated as the ratio of UA PI to MCA PI [9]. The SDP method was used to estimate amniotic fluid volume [16]. The AUCR was calculated as the ratio of SDP to UCR [10].

In our clinic, delivery is planned after the 37th week of pregnancy for patients diagnosed with preeclampsia who do not exhibit severe preeclampsia features or signs of fetal distress. Composite adverse perinatal outcomes (CANOs) include the presence of at least one of the following adverse outcomes: 5th-minute APGAR score < 7, transient tachypnea of the newborn (TTN), respiratory distress syndrome (RDS), need for continuous positive airway pressure (CPAP), need for mechanical ventilation, neonatal intensive care unit (NICU) admission, neonatal hypoglycemia, need for phototherapy, intraventricular hemorrhage (IVH), and neonatal sepsis.

### Statistical Analysis

Statistical analyses were performed using IBM SPSS Statistics version 26.0 (IBM Corporation, Armonk, NY, USA). The Kolmogorov–Smirnov test was used to assess the conformity of continuous variables to a normal distribution. Comparisons of continuous variables were performed using the Independent *t*-test for normally distributed data and the Mann–Whitney U test for non-normally distributed data. Descriptive statistics for continuous variables are presented as “mean ± standard deviation” for normally distributed data and as “median (min-max)” for non-normally distributed data. Categorical variables were compared using the Chi-squared test or Fisher’s exact test, as appropriate. Receiver operating characteristic (ROC) curve analysis was employed to calculate and compare areas under the curve (AUCs) and to identify optimal cut-off values for predictive parameters. Statistical significance for all analyses was set at a *P*-value of less than 0.05.

The required sample size for the study was determined using the G-Power 3.1.9.7 software (University of Dusseldorf, Dusseldorf, Germany). The sample size was estimated using a Student’s Paired t-Test with an 80% power, a significance level of α = 0.05, and a medium Cohen effect size. Based on these parameters, the minimum sample size required to achieve adequate statistical power was calculated to be at least 46 patients for each group, ensuring the robustness of the study’s findings.

## 3. Results

The comparative analysis of demographic, laboratory, and perinatal outcome data between preeclampsia and control groups is shown in Table 1. Among the 74 PE patients, 39 were classified as having EOPE and 35 as LOPE. Maternal age, weight, and BMI values were significantly higher in the PE group compared to controls (*p* = 0.014, *p* < 0.001, and *p* < 0.001, respectively). Weight gain during pregnancy, gravida, and parity were similar between both groups. Laboratory findings showed no significant differences in hemoglobin levels, platelet counts, or fibrinogen levels between the groups. However, the PE group had higher levels of AST, ALT, and uric acid levels (*p* = 0.010, *p* = 0.002, and *p* < 0.001, respectively), while albumin was significantly lower (*p* < 0.001). Gestational age at delivery was earlier (median 36.4 weeks, range 29.4–39 weeks vs. median 38.5 weeks, range 34–39.2 weeks, *p* < 0.001) and preterm birth was more frequent (58.1% vs. 12.5%, *p* < 0.001) in the PE group compared to controls. Cesarean section was more common in the PE group (*p* = 0.004). Neonates born to mothers with preeclampsia were of similar gender ratio, but had lower birth weights compared with controls (2416 ± 749 g vs. 3108 ± 429 g, *p* < 0.001). The 1st- and 5th-minute APGAR scores were lower in the PE group (*p* < 0.001). Adverse neonatal outcomes, including TTN (21.6% vs. 5%, *p* = 0.003) and RDS (18.9% vs. 1.3%, *p* < 0.001), were more frequent in the PE group. Also, the need for CPAP (35.1% vs. 7.5%, *p* < 0.001) and mechanical ventilation (18.9% vs. 2.5%, *p* = 0.001) were more common in neonates born to PE mothers. NICU admissions were more frequent in the PE group (40.5% vs. 12.5%, *p* < 0.001). Neonatal hypoglycemia, phototherapy needs, IVH, and neonatal sepsis showed no significant differences between groups. CANOs were significantly higher in the PE group compared to controls (40.5% vs. 16.3%, *p* = 0.001). Perinatal mortality was not observed in either group (Table 1).

The comparative analysis of Doppler parameters between the control and preeclampsia groups is shown in Table 2. The UA S/D ratio and PI were significantly higher in the PE group compared to controls (*p* = 0.006 and *p* = 0.004, respectively). The UtA S/D ratio was elevated in the PE group compared to controls (*p* = 0.001). Although there was no significant difference in the UtA PI between the groups, the UtA PI/UA PI ratio was significantly higher in the PE group than in controls (*p* = 0.008). The MCA S/D ratio and PI were significantly lower in the preeclampsia group versus controls (*p* = 0.008 and *p* = 0.012, respectively). The MCA PI/UtA PI ratio was markedly lower in PE group compared to controls (*p* < 0.001). MCA PSV was similar in both groups. The CPR was significantly reduced in the PE group compared to controls (1.58 ± 0.5 vs. 1.9 ± 0.5, *p* < 0.001). The UCR was higher in PE group compared to controls (0.7 ± 0.25 vs. 0.56 ± 0.18, *p* < 0.001). Similarly, the CPUR (1.67 ± 1.02 vs. 2.56 ± 1.3 vs., *p* < 0.001) and AUCR (8.25 ± 3.37 vs. 10.1 ± 3.68, and *p* = 0.001) were significantly lower in PE cases compared to controls (Table 2).

The comparative analysis of Doppler parameters between EOPE and LOPE patients is shown in Table 3. The UA S/D ratio and PI were significantly higher in EOPE cases compared to LOPE cases (*p* = 0.005 and *p* = 0.022, respectively). The UtA S/D ratio and UtA PI were elevated in the EOPE group compared to LOPE group (*p* = 0.001 and *p* < 0.001, respectively). UtA PI/UA PI ratio was similar between the two groups. MCA PSV, MCA S/D ratio, and MCA PI were similar between groups. MCA PI/UtA PI was significantly higher in the LOPE group (*p* = 0.001). The CPR was significantly reduced in the EOPE group compared to LOPE group (1.47 ± 0.49 vs. 1.7 ± 0.49, *p* = 0.048). The UCR was higher in EOPE group compared to LOPE group (0.75 ± 0.25 vs. 0.64 ± 0.24, *p* = 0.049). The CPUR (1.28 ± 0.86 vs. 2.11 ± 1, *p* < 0.001) and AUCR (7.54 ± 3.36 vs. 9.03 ± 3.26, *p* = 0.048) were significantly lower in EOPE cases compared to LOPE cases (Table 3).

The comparative analysis of Doppler parameters in preeclampsia cases with and without composite adverse neonatal outcomes is presented in Table 4. The UA S/D ratio and PI were significantly higher in PE cases with CANO compared to those without CANO (*p* = 0.007 and *p* = 0.035, respectively). The UtA S/D ratio and PI were markedly elevated in cases with CANO compared to those without CANO (*p* = 0.004 and *p* = 0.023, respectively). The UtA PI/UA PI ratio, MCA PSV, MCA S/D ratio, and MCA PI were similar between groups. The MCA PI/UtA PI ratio was significantly lower in the CANO group (*p* = 0.013). The CPUR was significantly lower in cases with CANO compared to those without CANO (1.27 ± 0.73 vs. 1.96 ± 1.1, *p* = 0.004). Similarly, the AUCR was reduced in cases with CANO (7.2 ± 3.06 vs. 9.97 ± 3.42, *p* = 0.025). The UCR and CPR did not show statistically significant differences between the groups (Table 4).

**Figure 1:**
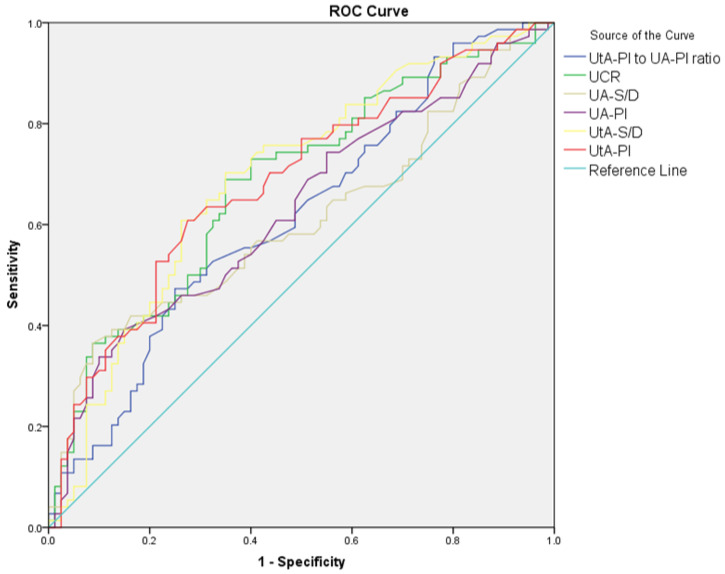
ROC curves comparing UA S/D, UA PI, UtA S/D, UtA PI, UtA PI/UA PI ratio, and UCR in distinguishing preeclampsia from control groups.

**Figure 2:**
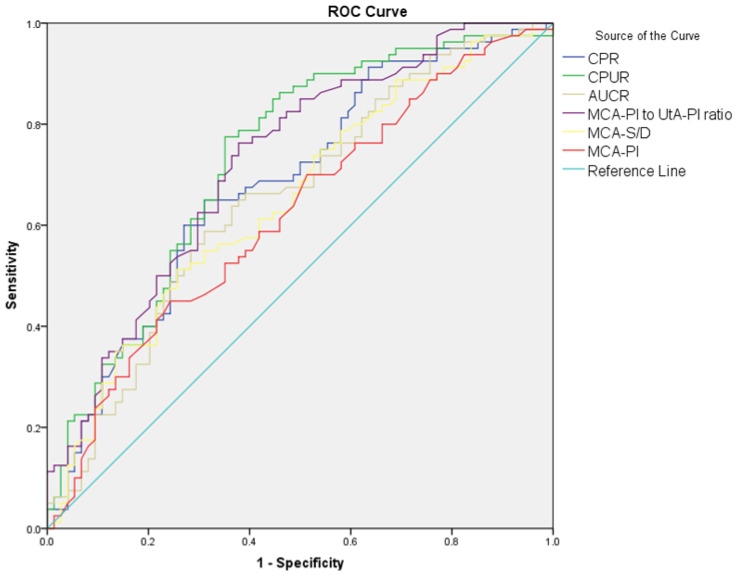
ROC curves comparing MCA S/D, MCA PI, MCA PI/UtA PI ratio, CPUR, AUCR, and CPR in distinguishing preeclampsia from control groups.

**Figure 3:**
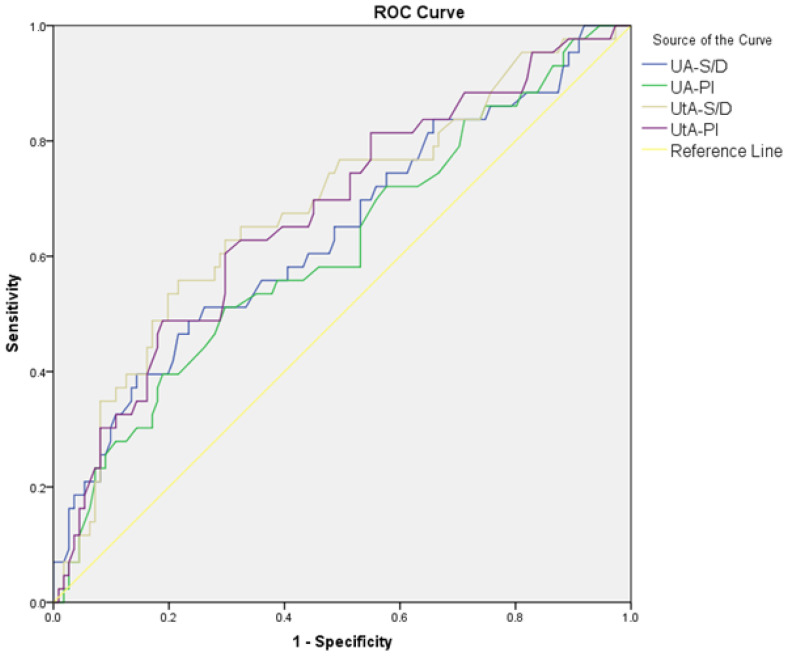
ROC curves comparing UA S/D, UA PI, UtA S/D ratio, and UtA PI in distinguishing composite adverse neonatal outcomes in the PE group.

**Figure 4:**
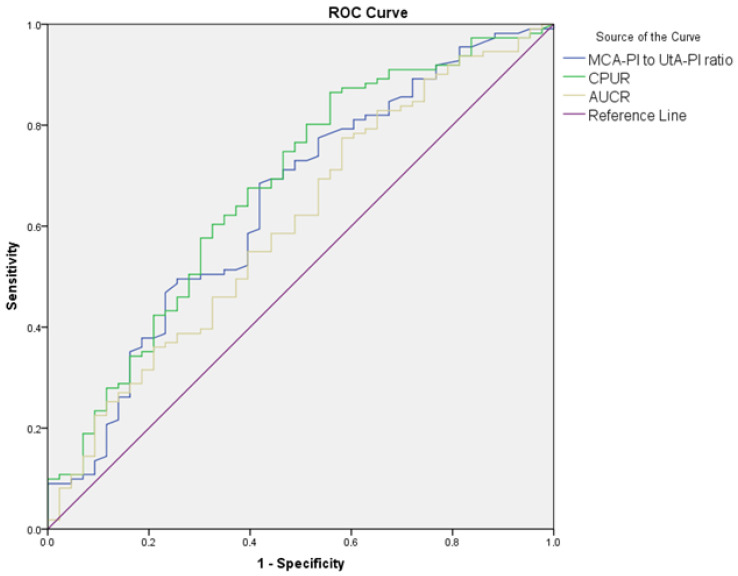
ROC curves comparing MCA PI/UtA PI ratio, CPUR, and AUCR in distinguishing composite adverse neonatal outcomes in the PE group.

The diagnostic performance of Doppler parameters for PE and composite adverse neonatal outcomes is summarized in Table 5. For PE diagnosis, the UA S/D ratio (AUC: 0.608, cut-off: >2.56, *p* = 0.021) showed limited diagnostic performance, while the UA PI performed slightly better (AUC: 0.630, cut-off: >0.90, *p* = 0.005). The UtA S/D ratio (AUC: 0.692, cut-off: >2.1, *p* < 0.001) and the UtA PI (AUC: 0.685, cut-off: >0.97, *p* < 0.001) also showed significant diagnostic performance. The MCA S/D ratio (AUC: 0.648, cut-off: <4.52, *p* = 0.002) and MCA PI (AUC: 0.620, cut-off: <1.56, *p* = 0.010) showed limited diagnostic performance, while the MCA PI/UtA PI ratio (AUC: 0.723, cut-off: <1.72, *p* < 0.001) demonstrated better accuracy. Among combined ratios, CPUR (AUC: 0.730, cut-off: <1.85, *p* < 0.001) showed the highest diagnostic accuracy for PE, followed by UCR (AUC: 0.683, cut-off: >0.57, *p* < 0.001), CPR (AUC: 0.683, cut-off: <1.77, *p* < 0.001), and AUCR (AUC: 0.652, cut-off: <8.54, *p* = 0.001). For composite adverse neonatal outcomes, the UA S/D ratio (AUC: 0.678, cut-off: >2.8, *p* = 0.010) and UA PI (AUC: 0.650, cut-off: >0.97, *p* = 0.029) showed diagnostic performance. The UtA S/D ratio (AUC: 0.701, cut-off: >2.61, *p* < 0.001) and UtA PI (AUC: 0.692, cut-off: >1.16, *p* = 0.005) demonstrated better diagnostic accuracy. Among combined ratios, CPUR (AUC: 0.705, cut-off: <1.25, *p* = 0.003) again provided the highest diagnostic accuracy for CANOs, followed by AUCR (AUC: 0.660, cut-off: <7.64, *p* = 0.020). The UCR and CPR could not significantly differentiate CANOs (Figure 1, Figure 2, Figure 3 and Figure 4).

## 4. Discussion

Identifying high-risk groups for PE, enabling early prediction of PE, and predicting adverse neonatal outcomes in PE cases are critical for effective patient management and postpartum maternal–fetal care, especially in the context of birth planning [11]. This study investigated the relationship between both conventional and novel combined Doppler parameters with PE diagnosis and adverse neonatal outcomes. The CPR, CPUR, and AUCR were significantly lower in the PE group compared to the control group, while the UCR was notably higher in the PE group. Among the combined ratios, the CPUR exhibited the highest diagnostic performance for both PE diagnosis and the prediction of CANOs. Additionally, while the UCR, CPR, and AUCR were significant for PE diagnosis, only AUCR demonstrated a significant association with the prediction of CANOs.

Placental dysfunction plays a critical role in the pathogenesis of PE. This dysfunction results in reduced placental blood flow, while simultaneously increasing cerebral blood flow, a compensatory mechanism believed to protect the fetal brain [17]. Doppler ultrasonography is a valuable tool for assessing these fetal adaptations and evaluating overall well-being [5,6]. Previous studies in PE patients have predominantly focused on conventional Doppler parameters, investigating the relationship between placental changes assessed through UtA Doppler parameters and fetal adaptations assessed through UA and MCA Doppler parameters [18,19,20,21]. However, in high-risk pregnancies such as PE, conventional Doppler parameters may not fully capture the complexity of fetal adaptations. Conventional Doppler findings alone are often limited to comprehensively assess all aspects of uteroplacental and fetal hemodynamics, leading to an increased interest in combined Doppler assessments. These combined parameters include the CPR, CPUR, UCR, and AUCR. Specifically, UCR and AUCR have not been investigated in PE before, to our knowledge.

The CPUR was first introduced by MacDonald et al. in 2018 as a novel predictor for late FGR [8]. CPUR is calculated by dividing the CPR, which represents the ratio of the MCA PI to the UA PI, by the UtA PI. This approach provides a comprehensive evaluation by incorporating Doppler parameters from the uterine, umbilical, and middle cerebral arteries. The first study of CPUR in EOPE was conducted by Oğuz et al., who reported significantly lower CPUR values in EOPE cases compared to controls. A CPUR value of ≤1.3652 demonstrated a sensitivity of 74.4% and a specificity of 94.9% for predicting EOPE. Additionally, lower CPUR values were observed in cases requiring NICU admission. However, in this study, LOPE cases were excluded and only NICU admission was analyzed as the adverse neonatal outcome [22]. Agaoglu et al. evaluated CPUR in a pregnancy-induced hypertension (PIH) group, including both gestational hypertension and preeclampsia cases, compared to controls. Their findings showed significantly lower CPUR values in the PIH group. Furthermore, in univariate analysis, the occurrence of CANO was six times higher in cases with low CPUR. An optimal CPUR cut-off value of 1.32 demonstrated 82% sensitivity and 79% specificity for predicting CANO (AUC: 0.826, *p* < 0.001). In their study, CANO was defined as the presence of any of the following criteria: NICU admission, a 5th-minute Apgar score of less than 7, and/or umbilical cord arterial pH below 7.10 [23]. In our study, both conventional and combined Doppler parameters were evaluated, and CPUR demonstrated the best diagnostic performance for both PE prediction and CANO prediction. For predicting PE, CPUR achieved an AUC of 0.730 (95% CI: 0.650–0.811) with a cut-off value of <1.85 (*p* < 0.001), sensitivity of 70%, specificity of 66.2%, a positive likelihood ratio (+LR) of 2.07, and a negative likelihood ratio (−LR) of 0.45. Similarly, CPUR showed strong diagnostic performance for predicting CANO, with an AUC of 0.705 (95% CI: 0.585–0.825), a cut-off value of <1.25 (*p* = 0.003), sensitivity of 65.9%, specificity of 66.7%, a +LR of 1.98, and a −LR of 0.51. These findings suggest that CPUR is a highly reliable parameter for predicting both PE and CANO in preeclampsia cases. In particular, our study stands out due to its evaluation of multiple conventional and novel combined Doppler parameters, comprehensive analysis of adverse neonatal outcomes, and inclusion of a homogeneous preeclampsia cohort encompassing both EOPE and LOPE cases.

The CPR is calculated as the ratio of the MCA PI to the UA PI. The CPR is considered to be a marker of centralization of fetal blood flow as an adaptation to placental insufficiency, such as that seen in PE. Regan et al. were the first to evaluate CPR in FGR cases for predicting PE, demonstrating that abnormal CPR was significantly associated with the subsequent development of severe PE. Women with abnormal CPR were 4.14 times more likely to develop severe PE, and the authors recommended that surveillance for the development of PE should be initiated when abnormal CPR is detected in FGR cases. Additionally, abnormal CPR was also 2.12 times more likely to be associated with a CANO [24]. Lodge et al. found that mean CPR was lower in pregnancies complicated by hypertensive disorders (chronic hypertension, pregnancy-induced hypertension, and preeclampsia) and was lowest in women with PE. Furthermore, low CPR in the PE group was 4.09 times more associated with a CANO [25]. Moawad et al. observed significantly lower CPR values lower in LOPE cases compared to controls, correlating CPR with birth weight but finding no significant relationship with 1- and 5-min APGAR scores or umbilical cord blood pH values [4]. Similarly, Zarean et al. investigated cases of preeclampsia or pregnancy-induced hypertension and found CPR to be associated with adverse neonatal outcomes, but the sensitivity and specificity for predicting these outcomes were relatively low [26]. In our study, <1.77 CPR could predict PE with 65% sensitivity and 68.9% specificity (AUC: 0.683, *p* < 0.001), but could not significantly predict CANO. These inconsistencies suggest that although CPR may indicate a diagnosis of PE, it may not provide sufficient safety or reliability for clinical decision-making alone and should be interpreted with caution in the context of adverse pregnancy outcomes.

The UCR, a reverse form of the CPR, has been widely studied in the context of FGR for predicting adverse perinatal outcomes. However, its application in PE cases has not been previously explored. Given the shared pathophysiological mechanisms of FGR and PE, this study aimed to investigate the diagnostic and predictive value of UCR in PE cases, specifically for PE diagnosis and CANO. In our study, >0.57 UCR could predict PE with 68.9% sensitivity and 65% specificity (AUC: 0.683, *p* < 0.001), but could not significantly predict CANO. The literature provides conflicting findings regarding the prediction of adverse neonatal outcomes by UCR. While some publications describe UCR as a better predictor than CPR [9,27], some publications have found no significant difference [28]. Mascio et al. specifically evaluated UCR in FGR cases and found that its predictive value for CANO was limited, with an AUC of only 0.575 [29].

In cases of placental insufficiency, the UA PI increases, leading to the redistribution of fetal blood flow to prioritize cerebral perfusion as a brain-protective mechanism. While cerebral perfusion increases, blood flow to peripheral organs decreases, including the kidneys, which may reduce fetal urine production and subsequently decrease amniotic fluid levels [17]. Considering the relationship between amniotic fluid and Doppler, there is a trend towards new combinations of amniotic fluid measurement, which has an important place in the assessment of fetal well-being, and Doppler parameters. Stumpfe et al. first presented a new combined Doppler parameter in a retrospective study of FGR patients as the ratio of SDP to UCR, combining the Doppler parameters UA PI, MCA PI, and SDP. Their study reported that AUCR could predict adverse perinatal outcomes (APOs) with higher accuracy in small-for-gestational-age (SGA) fetuses at term [10]. Later, in a prospective study by Besimoglu et al. on fetuses diagnosed with FGR, AUCR was reported as the best predictive tool for APOs [30]. AUCR has not been investigated in PE cases before. In our study, for predicting PE, AUCR achieved an AUC of 0.652 (95% CI: 0.565–0.739) with a cut-off value of <8.54 (*p* = 0.001), sensitivity of 63.8%, specificity of 63.5%, a +LR of 1.75, and a −LR of 0.57. Similarly, AUCR showed diagnostic performance for predicting CANOs, with an AUC of 0.660 (95% CI: 0.533–0.787), a cut-off value of <7.64 (*p* = 0.020), sensitivity of 65.9%, specificity of 60%, a +LR of 1.65, and a −LR of 0.57. These findings suggest that AUCR is a highly reliable parameter for predicting both PE and CANOs in preeclampsia cases.

Our study has some limitations. First, while we aimed to comprehensively assess neonatal outcomes, neonatal blood gas measurements could not be obtained for all patients, which may have limited the robustness of our analysis of CANOs. Second, the study was conducted at a single center, which ensured consistency in data collection but may limit the generalizability of our findings to broader populations. Research at multiple centers with diverse patient populations will increase the generalizability and external validity of these findings by incorporating genetic, environmental, and healthcare differences. Additionally, future research should focus on integrating these effective Doppler parameters into multivariable predictive models. These models could combine Doppler findings with clinical and biochemical markers to improve diagnostic accuracy and facilitate individualized risk assessments. Such advancements would allow for earlier interventions and more personalized management of preeclampsia. Despite these limitations, our study has several strengths. First, our study investigated conventional and combined Doppler parameters within the same patient group, allowing for direct comparisons of their diagnostic and predictive utility. In addition, our PE cohort included both EOPE and LOPE patients, enabling a detailed comparison of Doppler parameters between these two clinically distinct subgroups. Another important strength of our study is the investigation of relatively new parameters, including UCR and AUCR, in the context of PE and their association with adverse neonatal outcomes.

## 5. Conclusions

In this study, we investigated the relationship between conventional Doppler parameters, newly proposed combined Doppler ratios, and their utility in PE diagnosis and the prediction of CANO in PE patients. Among the combined ratios, the CPUR exhibited the highest diagnostic performance for both PE diagnosis and CANO prediction. Additionally, while the UCR, CPR, and AUCR were significant for PE diagnosis, only AUCR demonstrated a significant association with the prediction of CANOs. Identifying high-risk patients using these Doppler parameters may facilitate improved birth planning, prenatal care, and patient counseling, potentially enhancing outcomes for both mothers and neonates. However, further research involving multiple centers and larger sample sizes is essential to validate these findings and better understand the clinical benefits of incorporating AUCR and other advanced Doppler parameters into the management of preeclampsia. Also, future studies should also include patients with comorbid conditions, such as FGR, to provide a more comprehensive understanding of Doppler parameters in diverse clinical scenarios and to improve the applicability of these tools in routine obstetric practice.

## Figures and Tables

**Table 1:** Comparative analysis of demographic, laboratory, and perinatal outcome data between preeclampsia and control groups.

Parameter	Control n = 80 (51.9%)	EOPE n = 39 (25.3%)	LOPE n = 35 (22.7%)	Preeclampsia n = 74 (48.1%)	*p*-Value
Age (years)	28.1 ± 5	30.2 ±5.8	30.4 ± 5.9	30.3 ± 5.8	0.014
Weight (kg)	74.9 ± 11.1	85.9 ± 16	91.5 ± 19	88.5 ± 17.8	<0.001
Weight gain during pregnancy (kg)	10 (4–40)	10 (1–21)	10 (2–25)	10 (1–25)	0.312
BMI (kg/m^²^)	29 ± 4.3	33.1 ± 6.9	34.3 ± 6.8	33.6 ± 6.8	<0.001
Gravida	2 (1–6)	2 (1–6)	3 (0–6)	2 (0–6)	0.269
Parity	1 (0–4)	0 (0–5)	1 (0–5)	1 (0–5)	0.643
Nulliparity	43 (53.8%)	18 (46.2%)	22 (62.9%)	40 (54.1%)	0.970
Hemoglobin (g/dL)	11.5 ± 1.4	11.8 ± 1.3	11.5 ± 1.3	11.6 ± 1.3	0.613
Platelet count (10^9^/L)	237.3 ± 63.3	243 ± 78.2	223.6 ± 61.2	233.8 ± 70.8	0.749
AST (U/L)	17 ± 6.7	22.2 ± 12.7	21.1 ± 15.9	21.7 ± 14.2	0.010
ALT (U/L)	10.3 ± 5.5	16 ± 12	13.3 ± 10.3	14.7 ± 11.2	0.002
Albumin (g/L)	37.5 ± 3.4	34.4 ± 3.9	34.2 ± 3	34.3 ± 3.5	<0.001
Uric acid (mg/dL)	3.5 ± 0.9	5.2 ± 1.6	5.3 ± 1.4	5.27 ± 1.5	<0.001
Fibrinogen (mg/dL)	476.5 ± 86.4	512 ± 104.4	487.9 ± 103.6	500.6 ± 104	0.120
Gestational weeks at delivery (weeks)	38.5 (34–39.2)	34.3 (29.4–39)	37 (34–38.5)	36.4 (29.4-39)	<0.001
Preterm birth	10 (12.5%)	28 (71.8%)	15 (42.9%)	43 (58.1%)	<0.001
Delivery type					0.004
Cesarean section	45 (56.3%)	31 (79.5%)	27 (77.1%)	58 (78.4%)	
Vaginal birth	35 (43.8%)	8 (20.5%)	8 (22.9%)	16 (21.6%)	
Gender					0.068
Female	38 (47.5%)	30 (76.9%)	16 (45.7%)	46 (62.2%)	
Male	42 (52.5%)	9 (23.1%)	19 (54.3%)	28 (37.8%)	
Fetal weight (g)	3108 ± 429	2119 ± 766	2747 ± 580	2416 ± 749	<0.001
1st minute APGAR score	9 (5–9)	8 (2–9)	8 (6–9)	8 (2–9)	<0.001
5th minute APGAR score	10 (8–9)	9 (6–10)	10 (6–10)	9 (5–10)	<0.001
5th minute APGAR score < 7	0 (0%)	8 (20.5%)	3 (8.6%)	11 (14.9%)	<0.001
TTN	4 (5%)	11 (28.2%)	5 (14.3%)	16 (21.6%)	0.003
RDS	1 (1.3%)	12 (30.8%)	2 (5.7%)	14 (18.9%)	<0.001
Need for CPAP	6 (7.5%)	20 (51.3%)	6 (17.1%)	26 (35.1%)	<0.001
Need for mechanical ventilator	2 (2.5%)	11 (28.2%)	3 (8.6%)	14 (18.9%)	0.001
NICU admission	10 (12.5%)	22 (56.4%)	8 (22.9%)	30 (40.5%)	<0.001
Neonatal hypoglycemia	1 (1.3%)	1 (2.6%)	0 (0%)	1 (1.4%)	0.956
Need for phototherapy	1 (1.3%)	3 (7.7%)	1 (2.9%)	4 (5.4%)	0.196
IVH	1 (1.3%)	0 (0%)	0 (0%)	0 (0%)	0.999
Neonatal sepsis	0 (0%)	3 (7.7%)	1 (2.9%)	4 (5.4%)	0.051
Composite adverse neonatal outcomes *	13 (16.3%)	19 (48.7%)	11 (31.4%)	30 (40.5%)	0.001
Perinatal mortality	0 (0%)	0 (0%)	0 (0%)	0 (0%)	N/A

Abbreviations: EOPE: early-onset preeclampsia, LOPE: late-onset preeclampsia, BMI: body mass index, AST: aspartate aminotransferase, ALT: alanine aminotransferase, TTN: transient tachypnea of the newborn, RDS: respiratory distress syndrome, CPAP: continuous positive airway pressure, NICU: neonatal intensive care unit, IVH: intraventricular hemorrhage. Data are shown as mean ± standard deviation or median (minimum-maximum) or n, %. * Composite adverse neonatal outcomes include presence of at least one of the adverse outcomes: 5th-minute APGAR score < 7, TTN, RDS, need for CPAP, need for mechanical ventilation, NICU admission, neonatal hypoglycemia, need for phototherapy, IVH, and neonatal sepsis.

**Table 2:** Comparison of the doppler parameters of preeclampsia and control groups.

Parameter	Control n = 80 (51.9%)	Preeclampsia n = 74 (48.1%)	*p*-Value
SDP (mm)	52.7 ± 10.7	52.2 ± 13.9	0.825
UA S/D	2.5 ± 0.4	2.87 ± 1.1	0.006
UA PI	0.87 ± 0.19	0.97 ± 0.21	0.004
UtA S/D	2.24 ± 0.89	2.74 ± 1.03	0.001
UtA PI	2.73 ± 12.23	1.16 ± 0.54	0.273
UtA PI/UA PI	0.99 ± 0.47	1.24 ± 0.68	0.008
MCA PSV (cm/s)	53.08 ± 36.9	47.97 ± 10.2	0.262
MCA S/D	5.13 ± 1.61	4.41 ± 1.72	0.008
MCA PI	1.61 ± 0.35	1.47 ± 0.36	0.012
MCA PI/UtA PI	2.25 ± 1.07	1.52 ± 0.77	<0.001
UCR	0.56 ± 0.18	0.7 ± 0.25	<0.001
CPUR	2.56 ± 1.3	1.67 ± 1.02	<0.001
AUCR	10.1 ± 3.68	8.25 ± 3.37	0.001
CPR	1.9 ± 0.5	1.58 ± 0.5	<0.001

Abbreviations: SDP: single deepest pocket, UA: umbilical artery, S/D: systolic/diastolic ratio, PI: pulsatility index, UtA: uterine artery, MCA: middle cerebral artery, PSV: peak systolic velocity, UCR: umbilical-to-cerebral ratio, CPUR: cerebral–placental–uterine ratio, AUCR: amniotic-to-umbilical-cerebral ratio, CPR: cerebroplacental ratio. Data are shown as mean ± standard deviation.

**Table 3:** Comparison of the doppler parameters of EOPE and LOPE groups.

Parameter	EOPE n = 39 (25.3%)	LOPE n = 35 (22.7%)	*p*-Value
SDP (mm)	51.1 ± 14.6	53.4 ± 13.3	0.488
UA S/D	3.21 ± 1.36	2.5 ± 0.53	0.005
UA PI	1.02 ± 0.19	0.91 ± 0.22	0.022
UtA S/D	3.12 ± 1.14	2.32 ± 0.71	0.001
UtA PI	1.37 ± 0.57	0.93 ± 0.39	<0.001
UtA PI/UA PI	1.38 ± 0.74	1.08 ± 0.57	0.054
MCA PSV (cm/s)	45.8 ± 8.7	50.2 ± 11.3	0.072
MCA S/D	4.34 ± 1.86	4.48 ± 1.57	0.733
MCA PI	1.45 ± 0.37	1.48 ± 0.34	0.717
MCA PI/UtA PI	1.24 ± 0.64	1.84 ± 0.79	0.001
UCR	0.75 ± 0.25	0.64 ± 0.24	0.049
CPUR	1.28 ± 0.86	2.11 ± 1	<0.001
AUCR	7.54 ± 3.36	9.03 ± 3.26	0.048
CPR	1.47 ± 0.49	1.7 ± 0.49	0.048

Abbreviations: EOPE: early-onset preeclampsia, LOPE: late-onset preeclampsia, SDP: single deepest pocket, UA: umbilical artery, S/D: systolic/diastolic ratio, PI: pulsatility index, UtA: uterine artery, MCA: middle cerebral artery, PSV: peak systolic velocity, UCR: umbilical-to-cerebral ratio, CPUR: cerebral–placental–uterine ratio, AUCR: amniotic-to-umbilical-cerebral ratio, CPR: cerebroplacental ratio. Data are shown as mean ± standard deviation.

**Table 4:** Comparison of Doppler parameters in preeclampsia cases with and without composite adverse neonatal outcomes.

Parameter	With CANO * n = 30 (40.5%)	Without CANO * n = 44 (59.5%)	*p*-Value
SDP (mm)	48.8 ± 12	54.6 ± 14.8	0.079
UA S/D	3.29 ± 1.51	2.59 ± 0.57	0.007
UA PI	1.03 ± 0.2	0.93 ± 0.21	0.035
UtA S/D	3.15 ± 0.92	2.46 ± 1.02	0.004
UtA PI	1.33 ± 0.48	1.04 ± 0.55	0.023
UtA PI/UA PI	1.34 ± 0.58	1.18 ± 0.74	0.314
MCA PSV (cm/s)	48.5 ± 8.5	47.6 ± 11.4	0.738
MCA S/D	4.32 ± 1.47	4.48 ± 1.89	0.693
MCA PI	1.47 ± 0.39	1.47 ± 0.35	0.976
MCA PI/UtA PI	1.26 ± 0.64	1.71 ± 0.81	0.013
UCR	0.76 ± 0.27	0.67 ± 0.23	0.143
CPUR	1.27 ± 0.73	1.96 ± 1.1	0.004
AUCR	7.2 ± 3.06	9.97 ± 3.42	0.025
CPR	1.47 ± 0.45	1.66 ± 0.53	0.105

Abbreviations: CANO: composite adverse neonatal outcome, SDP: single deepest pocket, UA: umbilical artery, S/D: systolic/diastolic ratio, PI: pulsatility index, UtA: uterine artery, MCA: middle cerebral artery, PSV: peak systolic velocity, UCR: umbilical-to-cerebral ratio, CPUR: cerebral–placental–uterine ratio, AUCR: amniotic-to-umbilical-cerebral ratio, CPR: cerebroplacental ratio. Data are shown as mean ± standard deviation. * Composite adverse neonatal outcomes include presence of at least one of the following adverse outcomes: 5th-minute APGAR score < 7, TTN, RDS, need for CPAP, need for mechanical ventilation, NICU admission, neonatal hypoglycemia, need for phototherapy, IVH, and neonatal sepsis.

**Table 5:** Comparative diagnostic performance measures for Doppler parameters diagnosis and composite adverse neonatal outcome in the preeclampsia group.

Study Groups	Parameters	AUC (95% CI)	Cut-Off *	*p*	Sensitivity (%)	Specificity (%)	+LR	−LR
PE diagnosis	UA S/D	0.608 (0.517–0.699)	>2.56	0.021	56.8	58.8	1.38	0.73
	UA PI	0.630 (0.542–0.719)	>0.90	0.005	60.8	55	1.35	0.71
	UtA S/D	0.692 (0.608–0.776)	>2.1	<0.001	70.3	65	2.01	0.46
	UtA PI	0.685 (0.601–0.769)	>0.97	<0.001	60.8	72.5	2.21	0.54
	UtA PI/UA PI	0.618 (0.530–0.706)	>0.98	0.012	55.4	61.3	1.43	0.73
	MCA S/D	0.648 (0.561–0.734)	<4.52	0.002	61.3	58.1	1.46	0.67
	MCA PI	0.620 (0.532–0.709)	<1.56	0.010	58.8	58.1	1.40	0.71
	MCA PI /UtA PI	0.723 (0.643–0.803)	<1.72	<0.001	68.8	66.2	2.04	0.47
	UCR	0.683 (0.599–0.767)	>0.57	<0.001	68.9	65	1.97	0.47
	CPUR	0.730 (0.650–0.811)	<1.85	<0.001	70	66.2	2.07	0.45
	AUCR	0.652 (0.565–0.739)	<8.54	0.001	63.8	63.5	1.75	0.57
	CPR	0.683 (0.599–0.767)	<1.77	<0.001	65	68.9	2.09	0.51
Composite adverse neonatal outcomes *	UA S/D	0.678 (0.551–0.804)	>2.8	0.010	60	65.9	1.76	0.61
	UA PI	0.650 (0.521–0.779)	>0.97	0.029	66.7	65.9	1.96	0.51
	UtA S/D	0.701 (0.635–0.866)	>2.61	<0.001	70	75	2.8	0.4
	UtA PI	0.692 (0.569–0.815)	>1.16	0.005	63.3	77.3	2.8	0.5
	MCA PI /UtA PI	0.681 (0.558–0.805)	<1.21	0.008	65.9	63.3	1.8	0.54
	CPUR	0.705 (0.585–0.825)	<1.25	0.003	65.9	66.7	1.98	0.51
	AUCR	0.660 (0.533–0.787)	<7.64	0.020	65.9	60	1.65	0.57

AUC: area under curve; CI: confidence interval, LR: likelihood ratios, PE: preeclampsia, UA: umbilical artery, S/D: systolic/diastolic ratio, PI: pulsatility index, UtA: uterine artery, MCA: middle cerebral artery, UCR: umbilical-to-cerebral ratio, CPUR: cerebral–placental–uterine ratio, AUCR: amniotic-to-umbilical-cerebral ratio, CPR: cerebroplacental ratio. * Cut-off values were found according to Youden index. * Composite adverse neonatal outcomes include presence of at least one of the following adverse outcomes: 5th-minute APGAR score < 7, TTN, RDS, need for CPAP, need for mechanical ventilation, NICU admission, neonatal hypoglycemia, need for phototherapy, IVH, and neonatal sepsis.

## Data Availability

On reasonable request, the corresponding author will provide the information supporting this study’s conclusions.
